# Identifying the determinants of patient satisfaction in the context of antenatal care in Kenya, Tanzania, and Malawi using service provision assessment data

**DOI:** 10.1186/s12913-022-08085-0

**Published:** 2022-06-04

**Authors:** Kate Bergh, Sebawit Bishu, Henock B. Taddese

**Affiliations:** 1 School of Public Health, Faculty of Medicine, Imperial College London, Medical School Building, St Mary’s Hospital, Norfolk Place, London, W2 1PG UK; 2 Evans School of Public Policy and Governance, University of Washington Seattle, University of Washington, Box 353055, Seattle, WA 98195-3055 USA

**Keywords:** Patient satisfaction, Quality of care, Antenatal care, Women’s health, Sub-Saharan Africa

## Abstract

**Background:**

Antenatal care (ANC) is a service that can reduce the incidence of maternal and neonatal deaths when provided by skilled healthcare workers. Patient satisfaction is an important health system responsiveness goal which has been shown to influence adherence to healthcare interventions. This study aims to assess the determinants of pregnant women’s satisfaction with ANC across Kenya, Tanzania, and Malawi using nationally representative Service Provision Assessment data.

**Methods:**

Patient satisfaction was conceptualised mainly based on Donabedian’s theory of healthcare quality with patient characteristics, structure, and process as the major determinants. Bivariate and multivariate analyses were conducted to identify the potential determinants.

**Results:**

Findings show that satisfaction was negatively associated with women’s age (AOR: 0.95; 95% CI: 0.92–0.99) and having a secondary (AOR: 0.39; 95% CI: 0.17–0.87) or tertiary education (AOR: 0.41; 95% CI: 0.17–0.99) in Kenya. Women on their first pregnancy were more likely to report satisfaction in Tanzania (AOR: 1.62; 95% CI: 1.00–2.62) while women were less likely to report being satisfied in their second trimester in Malawi (AOR: 0.31; 95% CI: 0.09–0.97). The important structural and process factors for patient satisfaction included: private versus public run facilities in Kenya (AOR: 2.05; 95% CI: 1.22–3.43) and Malawi (AOR: 1.85; 95% CI: 0.99–3.43); level of provider training, that is, specialist versus enrolled nurse in Tanzania (AOR: 0.35; 95% CI: 0.13–0.93) or clinical technician in Malawi (AOR: 0.08; 95% CI: 0.01–0.36); and shorter waiting times across all countries.

**Conclusion:**

Findings highlight the importance of professional proficiency and efficient service delivery in determining pregnant women’s satisfaction with ANC. Future studies should incorporate both patient characteristics and institutional factors at health facilities into their conceptualisation of patient satisfaction.

**Supplementary Information:**

The online version contains supplementary material available at 10.1186/s12913-022-08085-0.

## Background

Maternal and neonatal mortality in sub-Saharan Africa (SSA) remains high with approximately 200,000 maternal deaths and over one million neonatal deaths per year [1, 2]. Antenatal care (ANC) is a service that can reduce the incidence of maternal and neonatal deaths when provided by skilled healthcare workers (HCWs) [3–5]. Since 2016, the World Health Organization (WHO) has updated its recommendation to a minimum of eight ANC visits for pregnant women; up from the longstanding recommendation of four ANC visits [6]. The ANC visits should be initiated in the first trimester and include a package of services: timely and relevant health information, screening for complications, tetanus toxoid (TT) vaccinations based on previous vaccine exposure, and daily iron and folic acid (IFA) tablets. However, Kenya, Tanzania and Malawi are still following the previously recommended guidelines for four ANC visits which is only being adhered to by 52% of women in SSA [7–10]. Sub-Saharan African countries will need to increase ANC utilisation to meet the third Sustainable Development Goal which aims to “ensure healthy lives and promote well-being for all at all ages” [11].

While improving health outcomes is the primary goal of a health system, the WHO also emphasises the importance of health system responsiveness goals such as improving patient satisfaction [12]. Patient satisfaction is an indicator of healthcare quality which may be influenced by both aspects of care (e.g., technical quality) as well as personal and environmental factors that influence the patients’ views prior to the healthcare experience, which are known as antecedents (e.g., general expectations) [13, 14]. Patient satisfaction has been linked to adherence to services in the context of Human Immunodeficiency Virus (HIV) and maternity care in SSA [15–17]. Thus, increasing satisfaction is bound to improve ANC attendance outcomes.

### Conceptualisation of patient satisfaction

Donabedian’s theory of healthcare quality defines patient satisfaction as a positive evaluation of the different aspects of the quality of care [18]. This model draws information from three indicators of healthcare quality: structure, process, and outcome. Structure refers to the organisational factors at the health facility (management, administration, and financing), physical attributes (infrastructure and equipment) and staffing (HCW availability and qualifications). Process includes the actions of the provider in diagnosing conditions and recommending treatment and the actions taken by patients in seeking and carrying out personal care. Outcomes refer to patient satisfaction. However, we argue that the model fails to capture the potential antecedents of patient satisfaction; a critique supported by Coyle et al. [19].

**Fig. 1:**
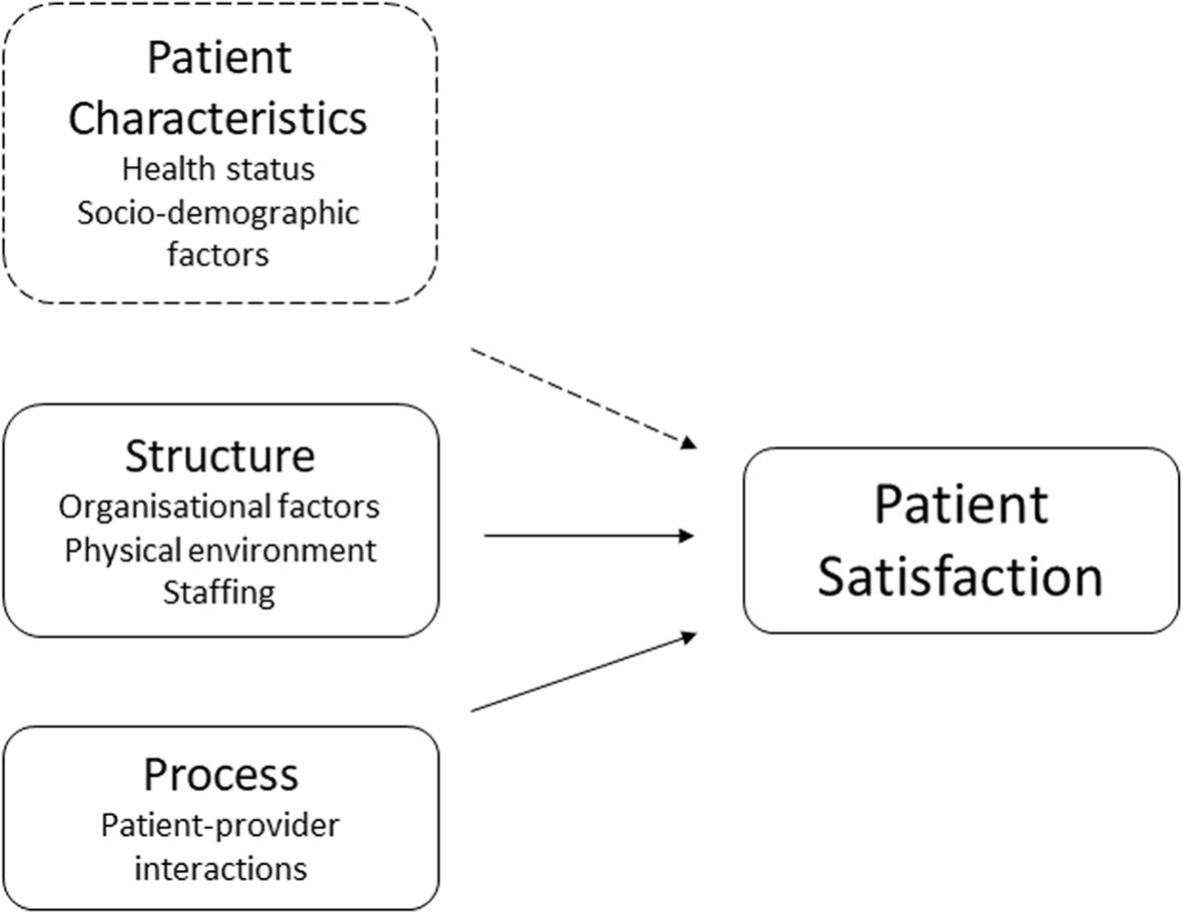
Guiding conceptual framework for assessing patient satisfaction: Adapted by integrating Donabedian’s conceptualisation with aspects of Fitzpatrick and Hopkins’ multiple models theory [18, 21, 22]

This study primarily uses Donabedian’s conceptualisation of patient satisfaction as a guiding framework as it is widely used and is considered the most comprehensive framework for patient satisfaction [18]. Donabedian highlights the importance of, and enumerates, the structural and process related factors that are critical for patient satisfaction. However, other theories such as the ‘value expectancy model’ describe how patients’ expectations may influence patient satisfaction; the ‘multiple models theory’ further explains that these expectations are in turn predicated by social and cultural factors [20, 21]. Furthermore, the ‘multiple models theory’ highlights the importance of ‘health status’ as yet another patient related factor [21]. We have thus adapted the Donabedian model to add a third overarching determinant of satisfaction called patient characteristics; to render our guiding framework a more holistic conception of patient satisfaction (Fig. 1) [18, 21, 22].

### Determinants of patient satisfaction

According to a systematic review by Batbaatar et al. (2017) which synthesised 108 international journal articles published between 1980 and 2014, the aspects of care with the greatest association with patient satisfaction, in order of importance, include: “interpersonal skills, competence, physical environment of the facility, accessibility, continuity of care, hospital characteristics, and outcome of care” [14]. However, the relationship between satisfaction and many socio-demographic characteristics remains inconclusive and may be specific to a particular country or grouping. Patient characteristics that have been shown to influence satisfaction in the ANC context in SSA include: distance to facility, marital status, income status, education, religion, prior children and health status [23–28]. However, the direction of these relationships is inconsistent across studies. In terms of structure, attending a private health facility, having regular external supervisory visits at the facility and good administration were positively associated with patient satisfaction in Kenya, as was having privacy during ANC consultations and appropriate ANC equipment in Nigeria [29–31]. A negative relationship between ANC provider training and satisfaction was reported in Namibia [29]. More generally, the WHO standards for maternal, newborn and child health provide eight domains for assessing the quality of care in a health system which are categorised into provision of care, experience of care and availability of resources [32, 33]. The domains relating to structure are evidence-based practices for routine care, actionable information systems, functioning referral systems, competent and motivated human resources and availability of essential physical resources.

According to Donabedian’s theory and the systematic review by Batbaatar et al. (2017), interpersonal relationships between patient and provider is the most important determinant of patient satisfaction [14, 18]. Respect, confidentially and communication between patient and provider have been highlighted as the key determinants of satisfaction in this regard in SSA [24, 25, 31, 34]. This is supported by the WHO standards for maternal, newborn and child health which include respect, preservation of dignity and protection of rights, emotional support and effective communication as key domains of quality of care [32, 33]. Furthermore, shorter waiting times are a consistent predictor of patient satisfaction in SSA and internationally [14, 29, 34]. There is also some evidence from Kenya, Ethiopia, Malawi and Nigeria to suggest that receiving medications, the number of clinical procedures performed and the number of ANC visits attended are positively associated with pregnant women’s satisfaction [25–27, 29, 31, 35].

To summarise, there is limited research on the determinants of patient satisfaction in the ANC context in SSA, variability in survey questionnaires affect comparability, and few studies have used a comprehensive conceptualisation of patient satisfaction which incorporate both patient characteristics and aspects of care. This study aims to identify the determinants of satisfaction in three sub-Saharan African countries (Kenya, Tanzania and Malawi) with similar geopolitcal contexts, socio-cultural norms and epidemiological traits. To this end, we analysed the Service Provision Assessment (SPA) data from the nationally representative Demographic and Health Survey (DHS) database across the three countries [36].

## Methods

### Setting

Three sub-Saharan African countries (Kenya, Tanzania, and Malawi) with available SPA data since 2010 were selected for this analysis due to their geopolitical, socio-cultural and epidemiological similarities. While this study does not statistically compare the determinants of satisfaction across the chosen countries, it offers a comparative analysis as far as providing descriptive statistics across all three countries; and thus, it was important that the countries included in the analysis were from similar contexts [37]. The Democratic Republic of Congo and Senegal were the only other sub-Saharan African countries with available SPA data since 2010.

Geopolitically, Kenya and Tanzania are both located in east Africa while Malawi is a nearby southern central African country. All three countries have a reasonably stable democracy and are classified as low or lower-middle income economies [38]. Socio-culturally, Christianity is the predominant religion in all three countries, while Islam also has a substantive representation and plays a major role in the socio-cultural landscape in Tanzania [39, 40]. From an epidemiological perspective, the prevalence of HIV and Malaria is very high in all three countries which is particularly risky for pregnant women and neonates [41, 42]. The average maternal mortality ratio (deaths per 100, 000 live births) is similar in Kenya and Malawi (342 vs. 349), although much higher in Tanzania (524) [1]. Maternal mortality rates are lower in most southern African countries (< 300). The average neonatal mortality ratio is similar across all three countries (19.8–21 per 1000 live births) as well as many other southern African countries [43].

Attendance of all four ANC visits is between 51 and 64% across the three countries [10]. In Kenya and Tanzania, most ANC consultations are provided by registered nurses with a three-year degree or diploma, and enrolled nurses with a two-year diploma, who work under the supervision of a registered nurse [44–46]. In Malawi, most ANC consultations are provided by medical assistants who have a two-year diploma in clinical medicine [47, 48]. In some cases, clinical technicians provide care in each country by performing diagnostic tests.

### Data

This study uses the most recent SPA data from Kenya (2010), Tanzania (2014–2015) and Malawi (2013–2014) available in the DHS database [36]. This data was accessed following an application to the DHS website (dhsprogram.com). The DHS Program has policies which ensure a high-quality dataset that is representative of the study population and ready for analysis. All DHS data collection procedure are compliant with the U.S. Department of Health and Human Services regulations for the protection of human subjects and country-specific laws and regulations.

Service Provision Assessments are health facility surveys which measure the quality and availability of basic health services, such as those for ANC, in a country. Surveys are standardised, although the questionnaires were updated in 2012, thus the Kenyan data was collected using an older version of the questionnaire. Nevertheless, the indicators available were almost identical across the three datasets with very few exceptions, which are highlighted where they influence any observed results. The assessment is comprised of four surveys:

1. The Facility Inventory Questionnaire gathers information on the availability of services, management systems, staffing, infrastructure, equipment, and medical supplies at the health facility.
2. The Provider Interview records the qualifications, experience, and perceptions of HCWs regarding health service delivery at the facility.
3. The ANC Observational Survey assesses the providers’ adherence to quality and health service delivery standards for ANC during consultation with a patient
4. The Client Exit Interview is conducted with clients following an observation of their ANC consultation and reports on the clients’ understanding of the services provided and perceptions of the quality of care received.

### Sampling

In each country, a regionally and nationally representative sample of public, private and faith-based facilities offering ANC services were randomly selected for the Facility Inventory Questionnaire [36]. As for the ANC Observational Surveys and Client Exit Interviews, these were carried out with an opportunistic sample of no more than 15 patients per facility and five per provider, based on the number of patients and providers present on the day of data collection for the Facility Questionnaire. This study utilised information from Facility, Observational and Exit Interviews. Facility and interview sampling is described for each country in Table 1 below [44, 45, 47].

**Table 1:** Health facility sampling by country

Sampling	Kenya	Tanzania	Malawi
Number of facilities nationwide	6192	6790	1060
Number of facilities selected for surveys	695	1188	977
Number of selected facilities offering ANC services	561	1031	643
Number of facilities with ANC observational and exit interview data	396	815	412
Number of ANC observations and exit interviews	1445	4010	2105

### Patient characteristics, structure and process

The SPA surveys provided ample indicators of patient characteristics, structure and process. Indicators of structure were selected from the Facility Inventory and Observational Questionnaires while indicators of process and patient characteristics were chosen from the ANC Observational and Exit Interviews. The selection of variables was guided by the conceptualisation of patient satisfaction adapted for this study (Fig. 1) as well as empirical evidence from a literature review of the determinants of patient satisfaction in the ANC context in SSA (Additional file 1: Appendix A). Composite additive or binary variables were created for certain aspects of structure and process; described in more detail in Table 2.

**Table 2:** Description of composite scores

Variable	Description
**Structure**	
Facility type (hospitals vs HCs/clinics)	Hospitals versus health centres (HCs), clinics and other smaller facilities (dispensaries, maternity unites and health posts)
Managing authority (public vs private)	Public facilities which include all government institutions versus private facilities which include private, faith-based, and non-governmental organisations
Basic amenities (score out of 5)	Electricity, running water, phone/radio, waiting area and latrine
ANC equipment (score out of 6)	Blood pressure machine, adult and foetal stethoscope, adult scale, examination bed and light
IFA and TT vaccines (score out of 2)	Iron and/or folic acid supplements and TT vaccines
Counselling (score out of 3)	Teaching aids, ANC guidelines and individual client card
**Process**	
Gave IFA and TT vaccines (score out of 2)	Iron and/or folic acid tablets and TT vaccines were provided
Provider explained purpose of IFA and TT vaccines (score out of 2)	Provider explained the purpose of the IFA tablets and TT vaccines
Number of procedures performed (score out of 10)	Took blood pressure, palpated abdomen for foetal presentation and height, listened for foetal heartbeat, weighed patient, examined conjunctiva/palms for anaemia, examined legs/feet/hands for oedema, examined patient’s breasts, conducted vaginal exam, and examined for swollen glands.
Infection control provided (yes/no)	Any testing, treatment or preventive measures taken against tapeworm infections, HIV or Malaria.

### Outcomes

The study applied a similar approach to measuring patient satisfaction as Do et al. (2017) which is a composite binary variable created using patient’s responses to 11 statements regarding common problems at the health facility [29]. Patients rated these problems as major, minor or no problem (Table 3). Clients were considered satisfied if they indicated no major problems with any of the 11 statements.

**Table 3:** Components of the composite variable for satisfaction [49]

Common problems at the healthcare facility	
1. Time you waited to see a provider	
2. Ability to discuss problems or concerns about your pregnancy	
3. Amount of explanation you received about the problem or treatment	
4. Privacy from having others see the examination	
5. Privacy from having others hear your consultation discussion	
6. Availability of medicines at this facility	
7. The hours of service at this facility, i.e., when they open and close	
8. The number of days services are available to you	
9. The cleanliness of the facility	
10. How the staff treated you	
11. Cost for services or treatments	

### Statistical analysis

The determinants of patient satisfaction were identified using a bivariate and multivariate analysis. The bivariate analysis was applied to test the independent association of patient characteristics and aspects of structure and process with patient satisfaction (Table 5). A logistic regression analysis was applied to test these associations. Independent variables with evidence of a significant association (*p*-value ≤ 0.05) in at least one country were included in the multivariate analysis for each country. Determinants of patient satisfaction highlighted in the systematic search (Additional file 1: Appendix A) which fitted into our conceptualisation of patient satisfaction were also included in the final model regardless of the results from the bivariate analysis. All patient characteristics were included in the final model to control for confounding, excluding urban/rural residence as this data was missing for Kenya.

A multivariate logistic regression analysis was performed for each country with patient satisfaction as the outcome variable (Table 6). A correlation analysis was conducted to ensure that no colinear variables (r = +/− 0.8) were included in the multivariate regression analysis. All descriptive statistics and analyses were adjusted by sample weight (PSU = facility, strata = region and facility type). Missing records were excluded from analyses. The number of missing records was less than 50 per variable.

## Results

### Patient characteristics

In general, patient characteristics were similar in Kenya, Tanzania, and Malawi (Table 4). Most women were between 20 and 35 years of age (≥ 69.72%) and had a primary school education (≥ 55.67%). However, the percentage of women with a tertiary education was notably higher in Kenya (10.91%) compared to Tanzania (1.75%) and Malawi (2.82%). Seventy percent or more of the study participants were multiparous and more than half were in their third trimester although this percentage was much higher in Kenya (71.19%) compared to Tanzania (51.61%) and Malawi (52.77%). Most women in Tanzania and Malawi lived in rural areas (72%). Finally, the percentage of women who attended the health facility nearest to their home was lower in Kenya (77.43%) compared to Tanzania (87.59%) and Malawi (90.1%). Attributes of structure and process are described in the supplementary information (Additional file 1: Appendix B and Appendix C).

**Table 4:** Patient characteristics of women who participated in the ANC observational and exit interviews

Variable	Kenya	Tanzania	Malawi
	N (%) (weighted)	N (%) (weighted)	N (%) (weighted)
Age			
< 20	195 (13.97)	761 (19.21)	406 (20.26)
20–35	1119 (80.05)	2762 (69.72)	1435 (71.51)
≥35	84 (5.98)	439 (11.07)	165 (8.23)
Level of education			
None	105 (7.48)	934 (23.33)	288 (13.95)
Primary	783 (55.67)	2415 (60.27)	1267 (61.31)
Secondary	364 (25.93)	587 (14.65)	453 (21.92)
Higher	153 (10.91)	69 (1.75)	58 (2.82)
Patient’s first pregnancy			
Yes	394 (28.54)	998 (25.06)	504 (24.47)
No	988 (71.46)	2987 (74.94)	1556 (75.53)
Trimester of pregnancy			
First trimester (≤ 12 weeks)	21 (1.50)	145 (3.64)	84 (4.10)
Second trimester (13–26 weeks)	384 (27.31)	1793 (44.75)	891 (43.13)
Third trimester (≥ 27 weeks)	1003 (71.19)	2067 (51.61)	1091 (52.77)
Previous stillbirth or infant mortality (< 1 week)			
Yes	281 (19.99)	724 (18.08)	508 (24.57)
No	1124 (80.01)	3282 (81.92)	1559 (75.43)
Facility nearest to home			
Yes	1088 (77.43)	3508 (87.59)	1860 (90.10)
No	317 (22.57)	497 (12.41)	204 (9.90)
Residence			
Urban	NA	1123 (28.05)	586 (28.38)
Rural	NA	2883 (71.95)	1481 (71.62)

*NA* in all fields of a cell means that this variable does not apply or is missing for the respective country

### Bivariate logistic regression findings

Table 5 describes the independent associations between potential determinants and patient satisfaction. Variables with a significant association with pregnant women’s satisfaction in at least one of the study countries included age, level of education, managing authority, system for recording client opinion, visual and auditory privacy, cleanliness, ANC provider training, waiting times before being seen by provider, provider inquiring about pregnancy-related problems and provision of infection control.

**Table 5:** Bivariate analysis of potential determinants of patient satisfaction in Kenya, Tanzania, and Malawi

Variable	Kenya		Tanzania		Malawi	
	AOR (95% CI)	*P*	AOR (95% CI)	*P*	AOR (95% CI)	*P*
**Patient Characteristics**						
Age (years)	0.97 (0.94–1.00)	0.10	0.99 (0.97–1.00)	0.30	0.98 (0.96–1.00)	0.24
Level of education (ref = none)	1.00		1.00		1.00	
Primary	0.37 (0.16–0.87)	0.02	1.10 (0.82–1.46)	0.51	0.89 (0.62–1.27)	0.54
Secondary	0.31 (0.13–0.76)	0.01	0.90 (0.63–1.30)	0.60	0.69 (0.44–1.06)	0.09
Tertiary	0.46 (0.19–1.06)	0.07	0.88 (0.44–1.76)	0.73	0.39 (0.16–0.93)	0.04
Patient’s first pregnancy (ref = no)	1.07 (0.74–1.55)	0.69	1.12 (0.90–1.40)	0.30	1.16 (0.91–1.48)	0.21
Trimester of pregnancy (ref = first)	1.00		1.00		1.00	
Second (13–26 weeks)	1.51 (0.52–4.34)	0.43	0.76 (0.45–1.28)	0.31	0.79 (0.42–1.45)	0.45
Third (≥ 27 weeks)	1.67 (0.60–4.65)	0.33	0.89 (0.53–1.49)	0.67	0.84 (0.45–1.56)	0.60
Previous stillbirth or infant mortality (< 1 week) (ref = no)	0.77 (0.48–1.24)	0.29	0.91 (0.70–1.19)	0.52	1.10 (0.80–1.49)	0.54
Facility nearest to home (ref = no)	1.17 (0.85–1.62)	0.31	1.10 (0.82–1.46)	0.51	1.21 (0.84–1.75)	0.30
Residence (ref = urban)	1.00		1.00		1.00	
Rural	NA	NA	1.17 (0.86–1.61)	0.30	0.96 (0.59–1.54)	0.87
**Structure**						
Organisational factors						
Facility type (ref = hospital)	1.00		1.00		1.00	
Health centre/clinic	0.99 (0.69–1.41)	0.98	1.01 (0.79–1.28)	0.92	0.81 (0.54–1.20)	0.30
Managing authority (ref = public)	1.00		1.00		1.00	
Private	2.42 (1.62–3.61)	< 0.01	1.39 (1.06–1.82)	0.02	1.07 (0.75–1.53)	0.69
Number of days for ANC per week (Kenya) or month (other)	0.91 (0.81–1.03)	0.15	0.99 (0.97–1.01)	0.81	0.99 (0.97–1.01)	0.69
Supervisory visit within the past six months (ref = no)	0.6 (0.24–1.47)	0.27	1.13 (0.55–2.29)	0.73	1.20 (0.77–1.85)	0.41
Monthly management meetings (ref = no)	0.73 (0.46–1.18)	0.21	1.03 (0.78–1.36)	0.81	1.19 (0.85–1.66)	0.29
Quality of care system (ref = no)	0.97 (0.65–1.45)	0.91	1.03 (0.80–1.33)	0.77	1.21 (0.85–1.72)	0.28
System for recording client opinion (ref = no)	0.97 (0.53–1.77)	0.94	0.90 (0.59–1.38)	0.66	1.93 (1.12–3.30)	0.02
Physical environment						
Basic amenities (score out of 5)	1.13 (0.87–1.48)	0.33	1.04 (0.92–1.17)	0.48	1.08 (0.91–1.28)	0.36
ANC equipment (score out of 6)	1.18 (0.95–1.47)	0.13	0.94 (0.79–1.13)	0.57	0.90 (0.76–1.07)	0.24
Available IFA and TT vaccines (score out of 3)	1.08 (0.85–1.38)	0.50	0.98 (0.84–1.14)	0.84	1.05 (0.88–1.26)	0.56
Counselling supplies (score out of 3)	1.02 (0.78–1.35)	0.83	0.97 (0.85–1.11)	0.76	0.98 (0.84–1.15)	0.86
Visual and auditory privacy (ref = no)	2.21 (1.01–4.43)	0.03	0.94 (0.52–1.69)	0.84	1.09 (0.54–2.22)	0.79
Clean (Kenya: ref. = no) (other: score out of 8)	0.75 (0.40–1.40)	0.38	1.08 (0.99–1.17)	0.05	1.09 (0.94–1.27)	0.22
Staffing						
HCWs available 24-hours per day (ref = no)	0.97 (0.31–3.01)	0.97	0.75 (0.56–1.02)	0.07	0.44 (0.12–1.53)	0.20
Sex of provider (male vs. female)	0.62 (0.37–1.02)	0.06	1.20 (0.82–1.76)	0.33	0.95 (0.68–1.33)	0.79
ANC provider training (ref = specialist)	1.00					
Clinical technician	NA	NA	NA	NA	0.14 (0.02–0.92)	0.04
Registered nurse with diploma	0.78 (0.50–1.23)	0.30	NA	NA	0.97 (0.13–6.84)	0.98
Enrolled nurse	0.68 (0.43–1.08)	0.11	0.54 (0.34–0.86)	0.01	0.70 (0.16–3.08)	0.65
**Process**						
Waiting time before being seen by provider (min)	0.98 (0.98–0.99)	< 0.01	0.99 (0.99–0.99)	< 0.01	0.99 (0.99–0.99)	< 0.01
Provider asked about pregnancy-related problems (ref = no)	1.14 (0.81–1.60)	0.42	1.25 (1.00–1.56)	0.04	1.11 (0.85–1.11)	0.42
Provider encouraged patient to ask questions (ref = no)	0.83 (0.56–1.22)	0.35	0.93 (0.71–1.23)	0.64	0.99 (0.71–1.38)	0.98
Provider discussed preparation for delivery (ref = no)	0.9 (0.71–1.14)	0.40	1.20 (0.91–1.59)	0.19	1.05 (0.69–1.58)	0.81
Gave IFA and TT vaccines (score out of 2)	1.07 (0.85–1.36)	0.53	1.02 (0.87–1.19)	0.79	0.93 (0.73–1.18)	0.59
Provider explained purpose of IFA and TT vaccines (score out of 2)	1.01 (0.80–1.27)	0.91	1.05 (0.91–1.22)	0.45	0.94 (0.75–1.18)	0.64
Procedures performed (score out of 10)	1.01 (0.90–1.13)	0.78	0.80 (0.96–1.08)	0.42	0.98 (0.90–1.06)	0.65
Infection control provided (ref = no)	0.67 (0.46–0.97)	0.04	1.08 (0.83–1.39)	0.55	1.06 (0.77–1.46)	0.68
Charged for services (ref = no)	1.20 (0.80–1.79)	0.36	0.80 (0.55–1.17)	0.27	0.79 (0.53–1.18)	0.26
Number of visits to this facility for this pregnancy	1.09 (0.96–1.23)	0.14	1.05 (0.96–1.14)	0.27	1.04 (0.95–1.15)	0.36

*AOR* adjusted odds ratio, *CI* confidence interval, *NA* in all fields of a cell means that this variable does not apply or is missing for the respective country

### Multivariate logistic regression findings

The association between patient characteristics and pregnant women’s satisfaction with ANC services are notably different in Kenya, Tanzania, and Malawi (Table 6). However, aspects of structure and process including managing authority, ANC provider training and waiting times had a more consistent relationship with satisfaction across the three countries.

**Table 6:** Multivariate logistic regression analysis of potential determinants of patient satisfaction in Kenya, Tanzania, and Malawi

Variable	Kenya		Tanzania		Malawi	
	AOR (95% CI)	*P*	AOR (95% CI)	*P*	AOR (95% CI)	*P*
**Patient Characteristics**						
Age (years)	0.95 (0.92–0.99)	0.03	1.01 (0.98–1.05)	0.26	1.02 (0.98–1.05)	0.28
Level of education (ref = none)	1.00		1.00		1.00	
Primary	0.59 (0.26–1.35)	0.22	0.90 (0.49–1.63)	0.73	1.09 (0.50–2.34)	0.82
Secondary	0.39 (0.17–0.87)	0.02	0.75 (0.37–1.54)	0.45	0.86 (0.36–2.06)	0.74
Higher	0.41 (0.17–0.99)	0.05	0.53 (0.24–1.19)	0.13	0.29 (0.06–1.40)	0.12
Patient’s first pregnancy (ref = no)	0.95 (0.60–1.48)	0.82	1.62 (1.00–2.62)	0.05	1.18 (0.67–2.07)	0.56
Trimester of pregnancy (ref = first)	1.00		1.00		1.00	
Second (13–26 weeks)	1.12 (0.37–3.38)	0.83	0.79 (0.32–1.92)	0.61	0.31 (0.09–0.97)	0.05
Third (≥ 27 weeks)	1.09 (0.36–3.32)	0.87	0.73 (0.29–1.79)	0.50	0.35 (0.11–1.07)	0.07
Previous stillbirth or infant mortality (< 1 week) (ref = no)	0.98 (0.55–1.74)	0.95	0.78 (0.48–1.27)	0.33	1.10 (0.63–1.91)	0.72
Facility nearest to home (ref = no)	1.38 (0.99–1.91)	0.05	0.79 (0.52–1.19)	0.26	0.97 (0.45–2.08)	0.95
**Structure**						
Organisational factors						
Managing authority (ref = public)	1.00		1.00		1.00	
Private	2.05 (1.22–3.43)	< 0.01	0.94 (0.57–1.54)	0.82	1.85 (0.99–3.43)	0.05
Supervisory visit within the past six months (ref = no)	1.14 (0.40–3.23)	0.79	1.10 (0.42–2.87)	0.83	1.26 (0.63–2.52)	0.50
System for recording client opinion (ref = no)	1.22 (0.71–2.11)	0.46	1.24 (0.92–1.68)	0.15	1.07 (0.80–1.45)	0.61
Physical environment						
ANC equipment (score out of 6)	0.84 (0.60–1.18)	0.31	0.97 (0.73–1.28)	0.86	0.67 (0.54–0.82)	< 0.01
Visual and auditory privacy (ref = no)	2.35 (0.94–5.88)	0.07	1.22 (0.60–2.49)	0.57	0.40 (0.04–3.91)	0.43
Clean (Kenya: ref. = no) (other: score out of 8)	0.68 (0.36–1.27)	0.23	1.07 (0.93–1.24)	0.32	1.13 (0.94–1.37)	0.18
Staffing						
Sex of provider (ref = no)	0.65 (0.30–1.38)	0.26	1.47 (0.85–2.55)	0.17	0.82 (0.41–1.63)	0.59
ANC provider training (ref = specialist)						
Clinical technician	NA	NA	NA	NA	0.08 (0.01–0.36)	< 0.01
Registered nurse with diploma	1.04 (0.36–3.00)	0.93	NA	NA	0.11 (0.01–1.05)	0.06
Enrolled nurse	1.12 (0.42–2.93)	0.81	0.35 (0.13–0.93)	0.04	0.34 (0.03–2.93)	0.33
**Process**						
Waiting time before being seen by provider (min)	0.98 (0.98–0.99)	< 0.01	0.99 (0.98–0.99)	< 0.01	0.99 (0.99–0.99)	0.05
Provider asked about pregnancy-related problems (ref = no)	1.20 (0.82–1.77)	0.33	1.12 (0.78–1.61)	0.53	0.89 (0.54–1.46)	0.65
Gave IFA and TT vaccines (score out of 2)	0.86 (0.60–1.25)	0.44	1.06 (0.81–1.40)	0.63	0.93 (0.60–1.44)	0.77
Explain purpose of IFA and TT vaccines (score out of 2)	1.22 (0.88–1.70)	0.22	0.80 (0.59–1.09)	0.17	1.07 (0.80–1.44)	0.63
Procedures performed (score out of 10)	0.95 (0.82–1.10)	0.57	1.03 (0.93–1.15)	0.47	0.94 (0.84–1.06)	0.35
Infection control provided (ref = no)	1.16 (0.67–2.01)	0.59	1.07 (0.70–1.66)	0.73	1.14 (0.59–2.21)	0.69
Charged for services (ref = no)	1.11 (0.68–1.81)	0.65	0.66 (0.37–1.14)	0.14	0.63 (0.25–1.56)	0.32
Number of visits to this facility for this pregnancy	1.09 (0.89–1.33)	0.38	0.89 (0.74–1.07)	0.23	1.07 (0.85–1.33)	0.54

*AOR* adjusted odds ratio, *CI* confidence interval, *NA* in all fields of a cell means that this variable does not apply or is missing for the respective country

In terms of patient characteristics, there was a negative association between satisfaction and women’s age (AOR: 0.95; 95% CI: 0.92–0.99; *P* < 0.05) as well as women with a secondary (AOR: 0.39; 95% CI: 0.17–0.87; *P* < 0.05) or tertiary education (AOR: 0.41; 95% CI: 0.17–0.99; *P* < 0.05) in Kenya. Satisfaction was positively associated with attendance of a facility nearest to home in Kenya with weak significance (AOR: 1.38; 95% CI: 0.99–1.91, *P* = 0.05), although a statistically non-significant (*P* > 0.25) negative association was observed in Tanzania and Malawi. Women on their first pregnancy were more likely to report being satisfied than multiparous women in Tanzania (AOR: 1.62; 95% CI: 1.00–2.62; *P* < 0.05) while pregnant women were less likely to report being satisfied in their second trimester compared to their first trimester in Malawi (AOR: 0.31; 95% CI: 0.09–0.97; *P* < 0.05).

Among the variables related to structure, attending a private rather than public health facility was associated with increased odds of satisfaction in Kenya (AOR: 2.05; 95% CI: 1.22–3.43, *P* < 0.01) and Malawi (AOR: 1.85; 95% CI: 0.99–3.43; *P* = 0.05). In terms of ANC provider training, pregnant women were less satisfied when attended by an enrolled nurse in Tanzania (AOR: 0.35; 95% CI: 0.13–0.93, *P* < 0.05), or a clinical technician (AOR: 0.08; 95% CI: 0.01–0.36, *P* < 0.01) and registered nurse with a diploma (AOR: 0.11; 95% CI: 0.01–1.05, *P* = 0.06) in Malawi, compared to a specialist medical doctor. The odds of satisfaction also decreased per item of ANC equipment available at facilities in Malawi (AOR: 0.67; 95% CI: 0.54–0.82, *P* < 0.01).

In terms of process, waiting time had a consistently negative association with satisfaction in all three countries (*P* < 0.05). No other aspect of process showed a statistically significant relationship with satisfaction.

## Discussion

The key findings of this study emphasise the importance of antecedents or patient characteristics that underlie pregnant women’s expectations of healthcare services as key determinants of satisfaction in Kenya, Tanzania, and Malawi; although differently across the three countries. Aspects of structure and process were more consistently associated with satisfaction across the three countries, including facility managing authority (public versus private), level of provider training and shorter waiting times.

### Patient characteristics

The negative association observed between age and satisfaction in Kenya is a surprising finding, as the majority of studies in the systematic review by Batbaatar et al. (2017) on the determinants of patient satisfaction in the international healthcare context have found the reverse to be true [14]. In the Kenyan case, it could be claimed that younger women may have less experience attending ANC visits for themselves or friends and family thereby lacking a benchmark by which to judge their experiences. However, it would seem more prudent to expect this relationship as being highly modulated by culture and other contextual factors. For example, Batbaatar et al. (2017) report a similar negative association from studies of satisfaction in former Soviet countries, suggesting that there might be cultural factors that undergird observed trends [14]. Batbaatar et al. (2017) further highlight some findings that suggest that relationships may not be linear, with more dynamic variabilities across different stages of a woman’s life.

There was also a negative relationship between pregnant women’s satisfaction and education in Kenya; this relationship has been inconsistent in the ANC context in SSA [26, 28]. The negative relationship between satisfaction and education was also observed by a study in Ethiopia whereby the authors explain it may be because educated women have higher expectations for ANC from being better informed [28]. The level of education was more varied in the Kenyan sample compared to Tanzania and Malawi which may have allowed the data to highlight this finding; therefore the same trend might have been observed in the other countries had the data included more women with higher education as in the Kenyan case.

In Tanzania, there was a positive association between satisfaction and being on your first pregnancy which agrees with previous studies from Rwanda and Tanzania [17, 24]. In Malawi, women in their second trimester were less satisfied compared to those in their first trimester, which is a novel finding. Women on their first pregnancy may have limited experience with ANC while women on their first trimester may have not interacted with the health system enough times to develop strong levels of dissatisfaction. In addition, women in their second trimester may feel physically and mentally different to women in their first trimester, which may influence their level of satisfaction thus highlighting the role of health status in determining patient satisfaction as described by the ‘multiple models theory’ [21].

These findings all suggest that women who have had a previous experience with ANC due to their age, a prior pregnancy, or a previous visit earlier on in their current pregnancy are less likely to be satisfied than women who had little or no experience with ANC before this visit. Furthermore, women with a higher level of education may know what to expect from ANC and are thus more difficult to satisfy. Physical and mental well-being may also influence pregnant women’s level of satisfaction, although further research is needed to explore the association between general health status and patient satisfaction. Therefore, patient satisfaction appears to be heavily influenced by patient characteristics and perceptions and expectations of the healthcare experience. Researchers and providers should consider a wide range of patient characteristics when assessing and interpreting satisfaction scores.

Additionally, patients were also more satisfied when attending a facility nearest to home in Kenya. These findings need to be gauged in view of other findings in the ANC context in SSA which found that patients travelling from more remote villages were more satisfied despite the longer journey; thereby highlighting the complexity of measuring satisfaction [14, 23, 27].

### Structure and process

In Kenya and Malawi, patients were more satisfied at private versus public health facilities. These findings are consistent with several other studies in ANC and family planning settings in SSA as well as the wider healthcare context [29, 50, 51]. These studies attribute their findings to shorter waiting times in the private sector and better hospitality. Waiting times were much shorter in the private versus public sector in this study.

In Tanzania and Malawi, patients were more satisfied when attended by specialist doctors compared to ANC providers with fewer qualifications. These findings contradict earlier findings from nationally representative studies in Kenya, Namibia and Malawi which used similar methodology and found no relationship and even a negative association in Namibia [23, 29]. However, in the wider healthcare context, there is strong evidence for a relationship between perceived competency of the healthcare provider and patient satisfaction [14]. Findings suggest that patients are not satisfied with the level of competency of enrolled nurses in Tanzania, and registered nurses with only a diploma or clinical technicians in Malawi. This is a significant problem in Tanzania where enrolled nurses serve almost half of all pregnant women attending ANC. Tanzania has very high rates of maternal mortality, making the task of upskilling nurses of utmost importance [1].

There was a surprisingly negative association between satisfaction and the amount of ANC equipment available at the health facility in Malawi. This result may be a reflection of the inadequacy of our measure for availability of equipment to indicate the readiness of the facilities to deliver good service.

Only major aspects of structure and process such as managing authority, provider training and waiting times were associated with patient satisfaction in this study. This finding is not altogether surprising as a review of the theoretical literature on patient satisfaction in 1994 by Williams argued that patients tend to express dissatisfaction when a major negative event occurs [52].

Nevertheless, these findings reinforce the importance of fundamental aspects of the healthcare system including proper management of health facilities, efficient service delivery and availability of skilled HCWs. Findings suggest that improving these components in health facilities will be central to improving patient satisfaction. Future policies should pay special attention to the discrepancies between public and private facilities, particularly in Kenya and Malawi, and may consider separating their analyses by managing authority to inform policies.

### Strengths and limitations

This analysis of data is guided by a contemporary conceptualisation of patient satisfaction which was based on an extensive review of the theoretical literature, as well as the empirical evidence in the ANC context in SSA. We used nationally representative SPA datasets across multiple country settings. Given the cross-sectional study design, the findings do not provide evidence for a causal relationship between determinants and satisfaction. Some factors related to socio-economic status, ethnicity and religion were not available for inclusion in this analysis but given the many other patient characteristics that were included in the final model, we are reassured that socio-demographic dimensions have been accounted for adequately. Finally, there is potential for a social desirability bias in ANC Observational Interview results as ANC consultations were conducted under observation by a data collector, but this bias should be uniform and ultimately not affect the findings.

## Conclusions

In conclusion, these findings underscore the importance of patient characteristics and prior experiences in influencing patient satisfaction, alongside structural and process factors. Future studies need to be comprehensive in incorporating this broad perspective and testing the interactions between personal, structural and process related factors. The findings highlight the importance of professional proficiency and management at heath facilities which further emphasise the importance of enhancing quality and efficiency of service provision across all types of facilities.

## Supplementary Information

**Additional file 1: Appendix A**. Systematic search strategy for journal articles from 2000 to 2020 relating to the determinants of patient satisfaction with ANC in SSA. **Appendix B**. Structural attributes at the health facilities analysed in Kenya, Tanzania, and Malawi. **Appendix C**. Attributes of structure and process reported in the ANC observational and exit interviews. **Appendix D**. Waiting time before being seen by providers in public and private health facilities.

## Data Availability

Data for this project can be accessed following an application to the DHS website (https://dhsprogram.com/). The datasets used included Kenya: SPA, 2010; Tanzania: SPA, 2014–2015; and Malawi: SPA, 2013–2014.

## Abbreviations

ANC: Antenatal care
AOR: Adjusted odds ratio
CI: Confidence interval
DHS: Demographic and Health Survey
HCW: Healthcare worker
HIV: Human Immunodeficiency Virus
IFA: Iron and folic acid
NA: Not applicable
PSU: Primary sampling unit
Ref: Reference
SPA: Service Provision Assessment
SSA: Sub-Saharan Africa
TT: Tetanus Toxoid
WHO: World Health Organization
