# Supplementary material for: Identifying the determinants of patient satisfaction in the context of antenatal care in Kenya, Tanzania, and Malawi using service provision assessment data
Source: BMC Health Serv Res. 2022 Jun 4;22:746. doi: 10.1186/s12913-022-08085-0 (PMC9167501; doi:10.1186/s12913-022-08085-0)
Supplement: Supplementary file 1 — Additional file 1: Appendix A. Systematic search strategy for journal articles from 2000 to 2020 relating to the determinants of patient satisfaction with ANC in SSA. Appendix B. Structural attributes at the health facilities analysed in Kenya, Tanzania, and Malawi. Appendix C. Attributes of structure and process reported in the ANC observational and exit interviews. Appendix D. Waiting time before being seen by providers in public and private health facilities. [file 12913_2022_8085_MOESM1_ESM.docx]

**Appendix**

**Appendix A** Systematic search strategy for journal articles from 2000 to 2020 relating to the determinants of patient satisfaction with ANC in SSA

| **Database** | **Key words** |
| --- | --- |
| Embase Classic + Embase | (predictor* OR determinant*OR factor*OR dimension* OR aspect* OR attribute* OR measurement*) AND (patient satisfaction OR perceived quality) AND (antenatal OR prenatal OR perinatal) AND (Africa* OR sub-Saharan Africa* OR Kenya* OR Tanzania* OR Malawi*) |
| Ovid Medline (R) All |  |
| Maternity & Infant Care Database (MIDIRS) |  |

**Appendix B** Structural attributes at the health facilities analysed in Kenya, Tanzania, and Malawi

| **Facility interviews** | **Kenya** | **Tanzania** | **Malawi** |
| --- | --- | --- | --- |
|  | **N (%) or mean (min-max) (weighted)** | | |
| **Structure** |  |  |  |
| Organisational factors |  |  |  |
| Facility type |  |  |  |
| Hospital | 121 (27.77) | 96 (9.94) | 263 (68.67) |
| Health centre/Clinic | 315 (72.23) | 875 (90.06) | 120 (31.33) |
| Managing authority |  |  |  |
| Public | 307 (70.37) | 794 (81.69) | 130 (34.07) |
| Private | 129 (29.63) | 177 (18.31) | 252 (65.93) |
| Number of days for ANC per week (Kenya) or month (other) | 4.32 (1-7) | 18.31 (1-30) | 15.5 (1-30) |
| Supervisory visit within the past six months | 412 (94.74) | 904 (93.06) | 346 (90.54) |
| Monthly management meetings | 263 (60.32) | 400 (41.47) | 256 (67.20) |
| Quality of care system | 172 (39.70) | 217 (22.46) | 159 (41.81) |
| System for recording client opinion | 183 (57.41) | 313 (68.05) | 147 (74.55) |
| Physical environment |  |  |  |
| Basic amenities (score out of 5) | 3.98 (1-5) | 2.87 (1-5) | 3.95 (1-5) |
| ANC equipment (score out of 6) | 4.96 (1-6) | 4.54 (1-6) | 4.67 (1-6) |
| Available IFA and TT vaccines (score out of 3) | 2.19 (0-3) | 1.59 (0-3) | 1.32 (0-3) |
| Counselling supplies (score out of 3) | 2.31 (0-3) | 1.17 (0-3) | 1.67 (0-3) |
| Visual and auditory privacy | 420 (96.40) | 920 (94.71) | 369 (96.34) |
| Clean (Kenya: %) (other: score out of 8) | 393 (90.19) | 6.67 (0-8) | 7.07 (0-8) |
| Staffing |  |  |  |
| HCWs available 24-hours per day | 243 (86.00) | 579 (59.62) | 375 (98.08) |

**Appendix C** Attributes of structure and process reported in the ANC observational and exit interviews

| **Observational and exit interviews** | **Kenya** | **Tanzania** | **Malawi** |
| --- | --- | --- | --- |
|  | **N (%) or mean (min-max) (weighted)** | | |
| **Structure** |  |  |  |
| Staffing |  |  |  |
| Sex of provider |  |  |  |
| Male | 360 (25.60) | 617 (15.40) | 514 (24.89) |
| Female | 1048 (74.40) | 3389 (84.60) | 1553 (75.11) |
| ANC provider training |  |  |  |
| Specialist | 38 (2.77) | 13 (0.34) | 10 (0.50) |
| Clinical technician | 5 (0.36) | NA | 11 (0.54) |
| Registered nurse (bsn) | 3.33 (0.24) | 797 (19.89) | 69 (3.34) |
| Registered nurse with diploma | 536 (38.69) | NA | 29 (1.41) |
| Enrolled nurse | 656 (47.34) | 1984 (49.53) | 374 (18.09) |
| **Process** |  |  |  |
| Waiting time before being seen by provider (min) | 73.39 (0-548) | 99.49 (0-600) | 124.03 (0-600) |
| Provider asked about pregnancy-related problems | 715 (51.38) | 2546 (65.18) | 1272 (61.71) |
| Provider encouraged patient to ask questions | 952 (67.59) | 3105 (77.59) | 1670 (80.89) |
| Provider discussed preparation for delivery | 778 (55.42) | 2987 (75.09) | 1777 (86.30) |
| Gave IFA and TT vaccines (score out of 2) | 0.99 (0-2) | 1.11 (0-2) | 1.15 (0-2) |
| Provider explained purpose of IFA and TT vaccines (score out of 2) | 0.67 (0-2) | 0.9 (0-2) | 0.78 (0-2) |
| Procedures performed (score out of 10) | 7.23 (0-10) | 5.84 (0-10) | 6.09 (0-10) |
| Infection control provided | 1168 (82.94) | 3175 (79.25) | 1681 (81.3) |
| Charged for services | 907 (64.82) | 395 (9.86) | 229 (11.10) |
| Number of visits to this facility for this pregnancy | 2.13 (1-5) | 1.95 (1-5) | 2.10 (1-5) |

**Appendix D** Waiting time before being seen by providers in public and private health facilities

| **Waiting time before being seen by provider (min)** | **Kenya** | **Tanzania** | **Malawi** |
| --- | --- | --- | --- |
|  | **N (min-max)** | **N (min-max)** | **N (min-max)** |
| Public | 83.12 (0-548) | 104.13 (0-600) | 134.25 (0-600) |
| Private | 43.93 (0-548) | 79.52 (0-600) | 95.30 (0-600) |
